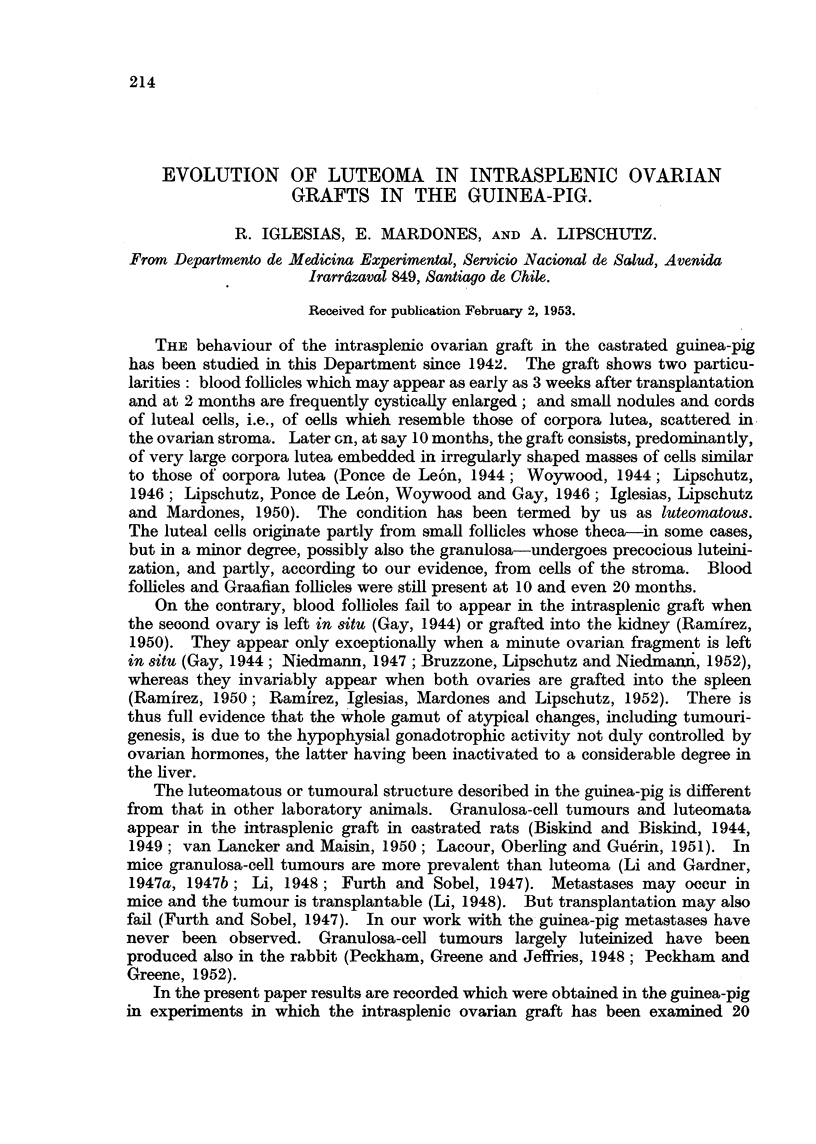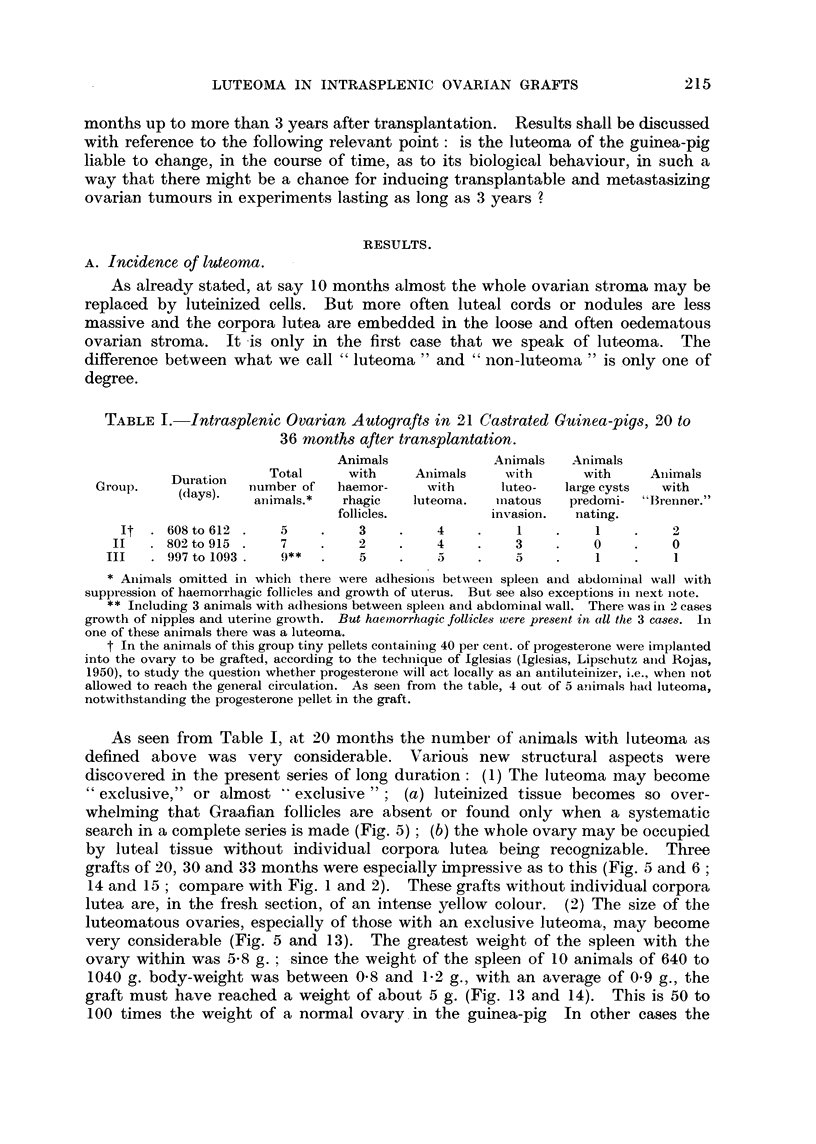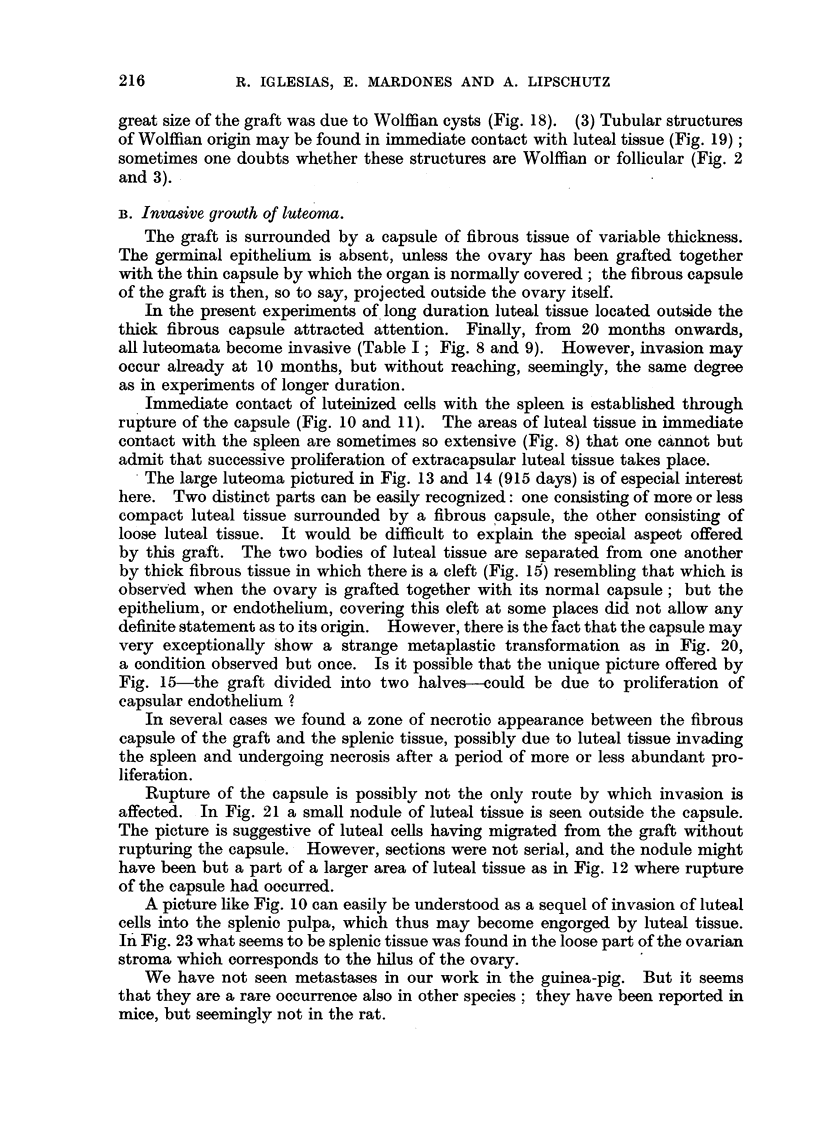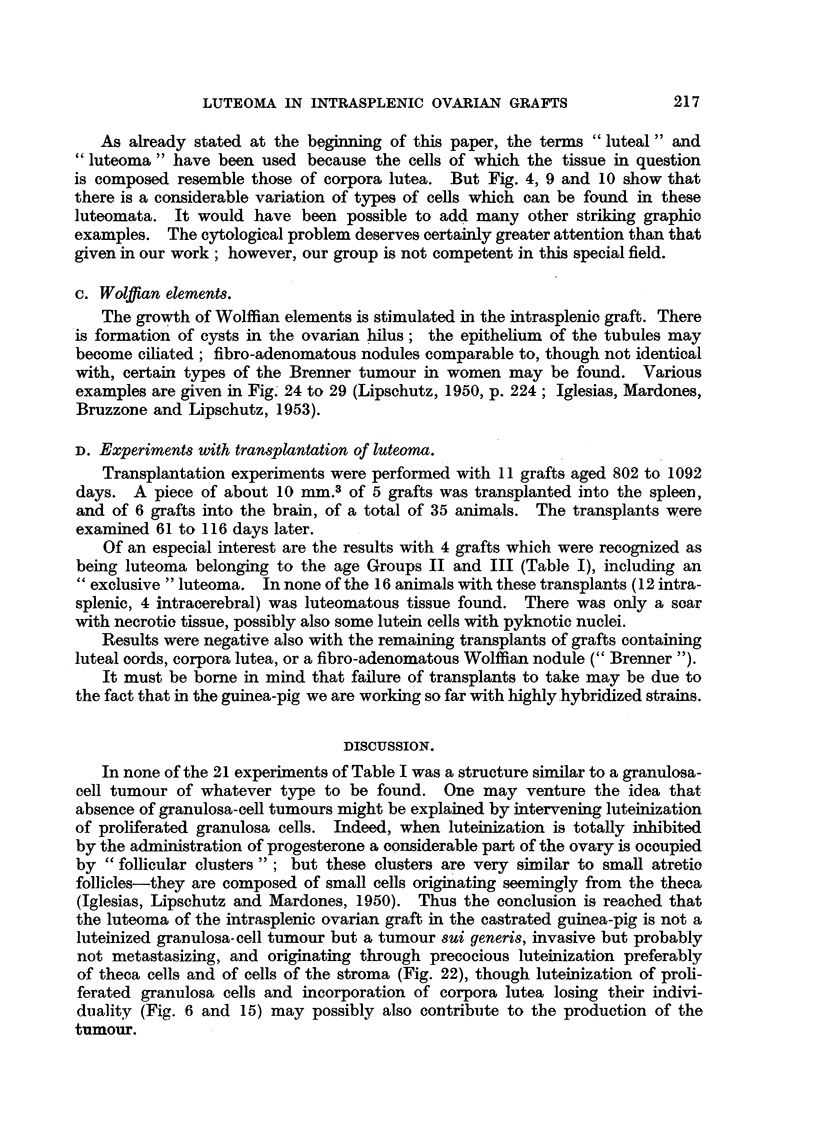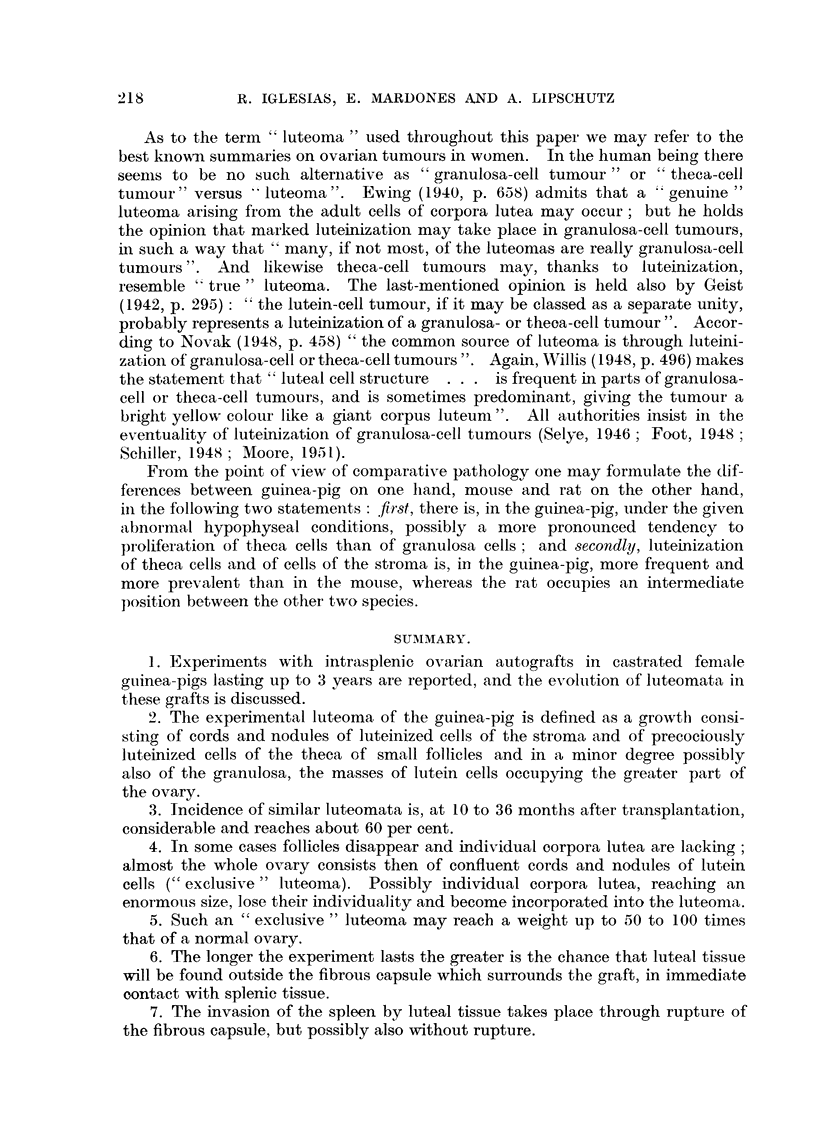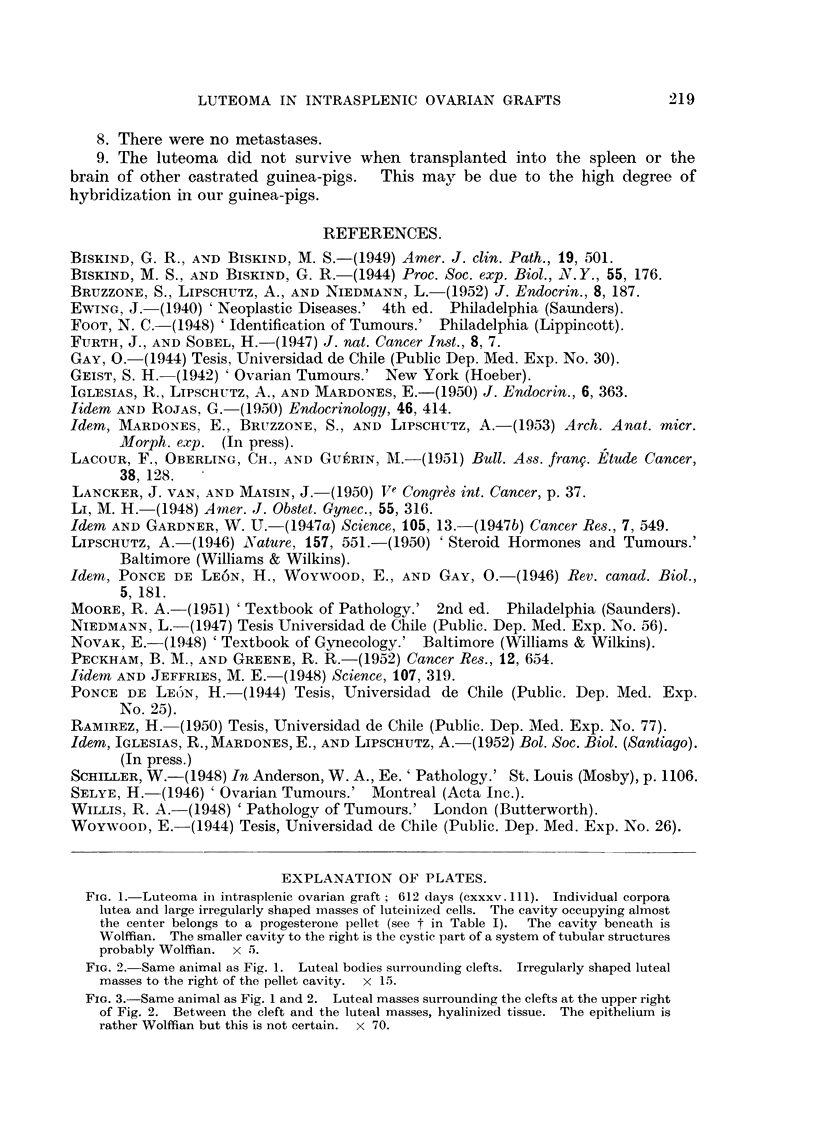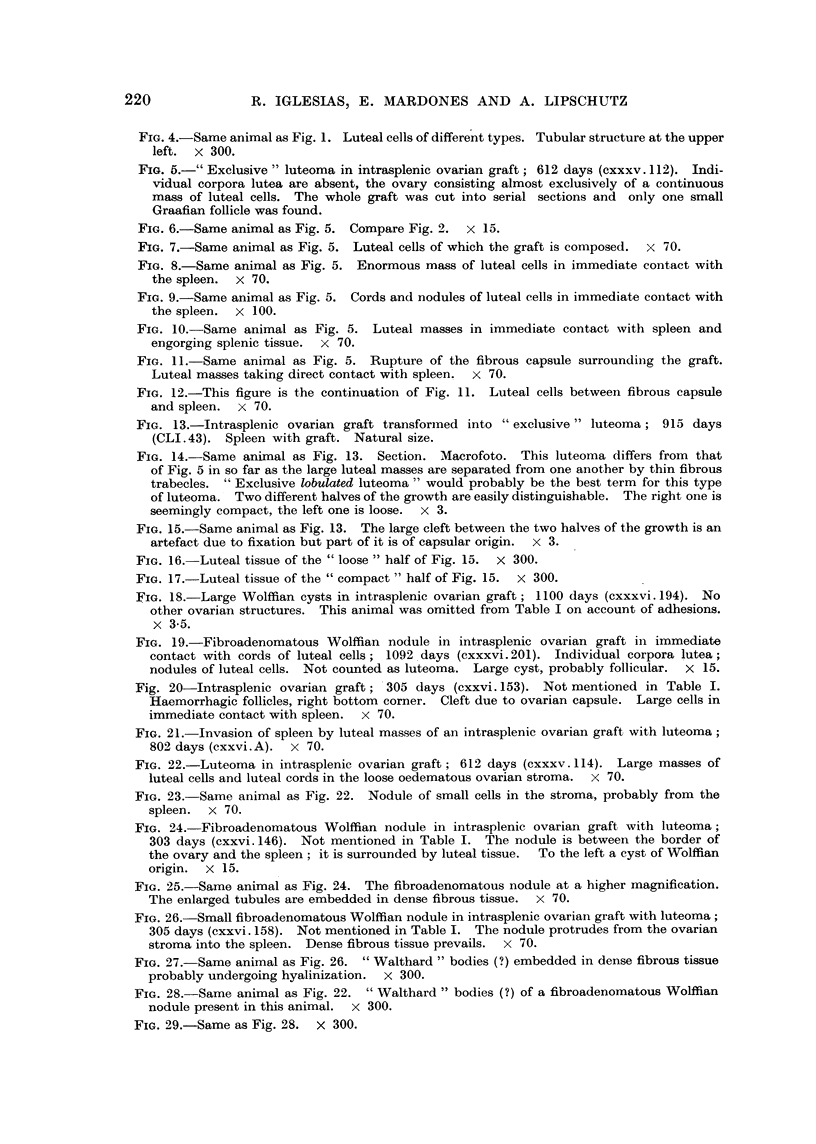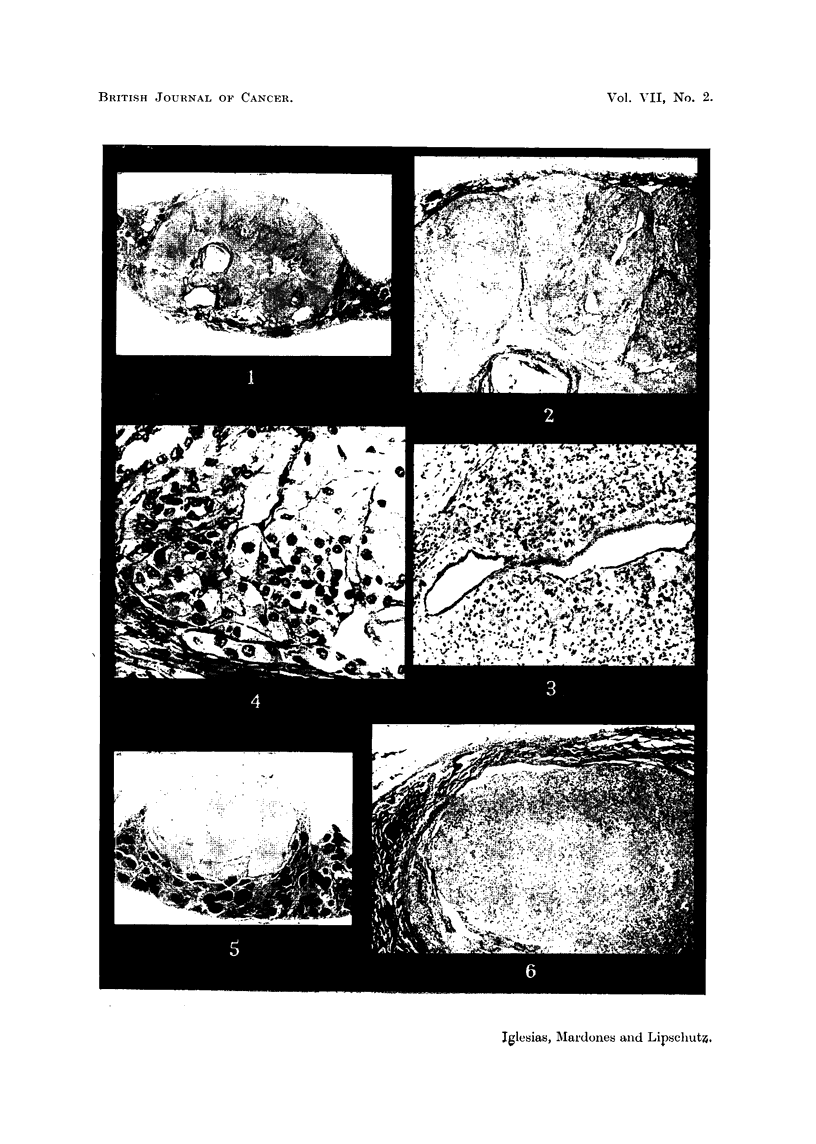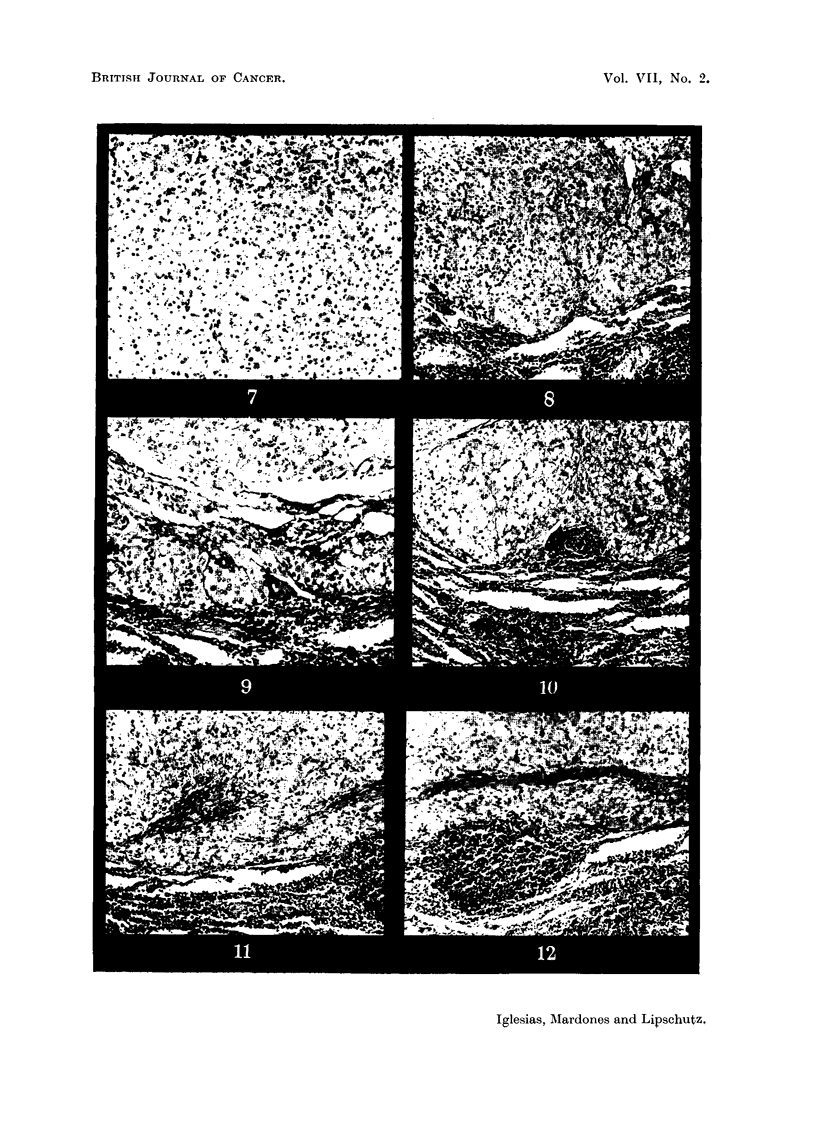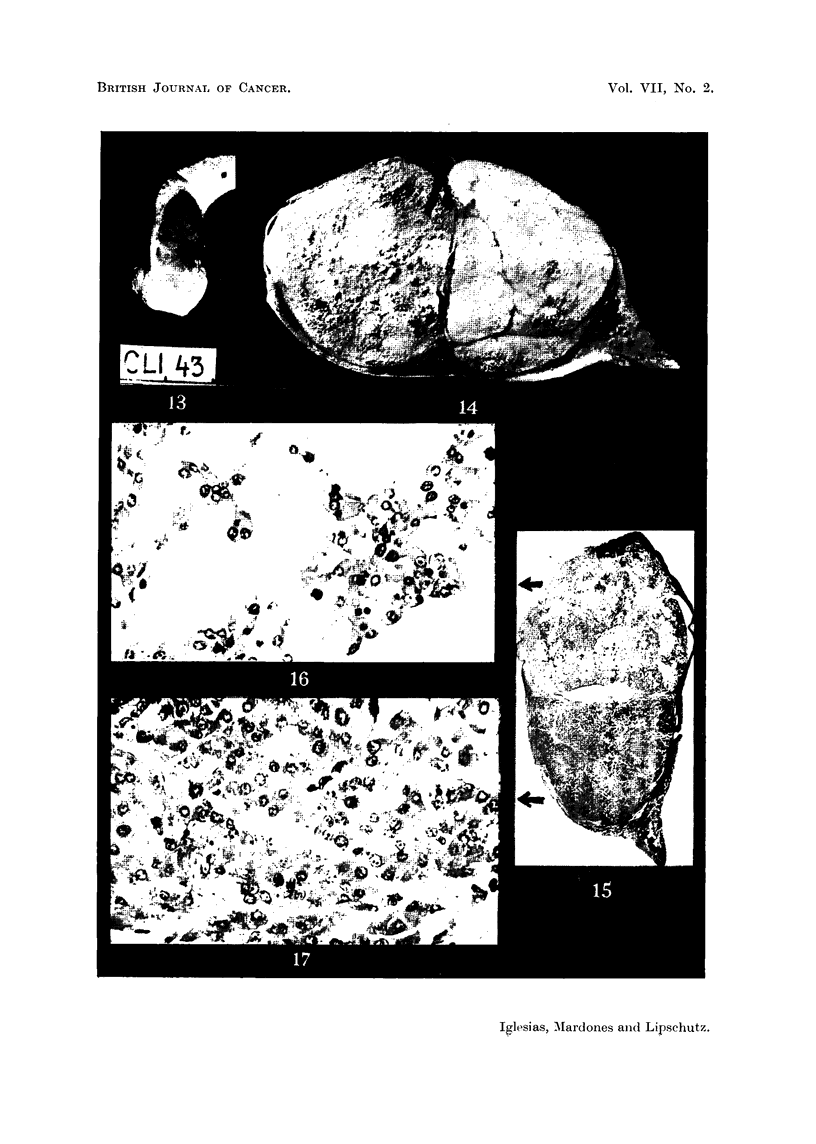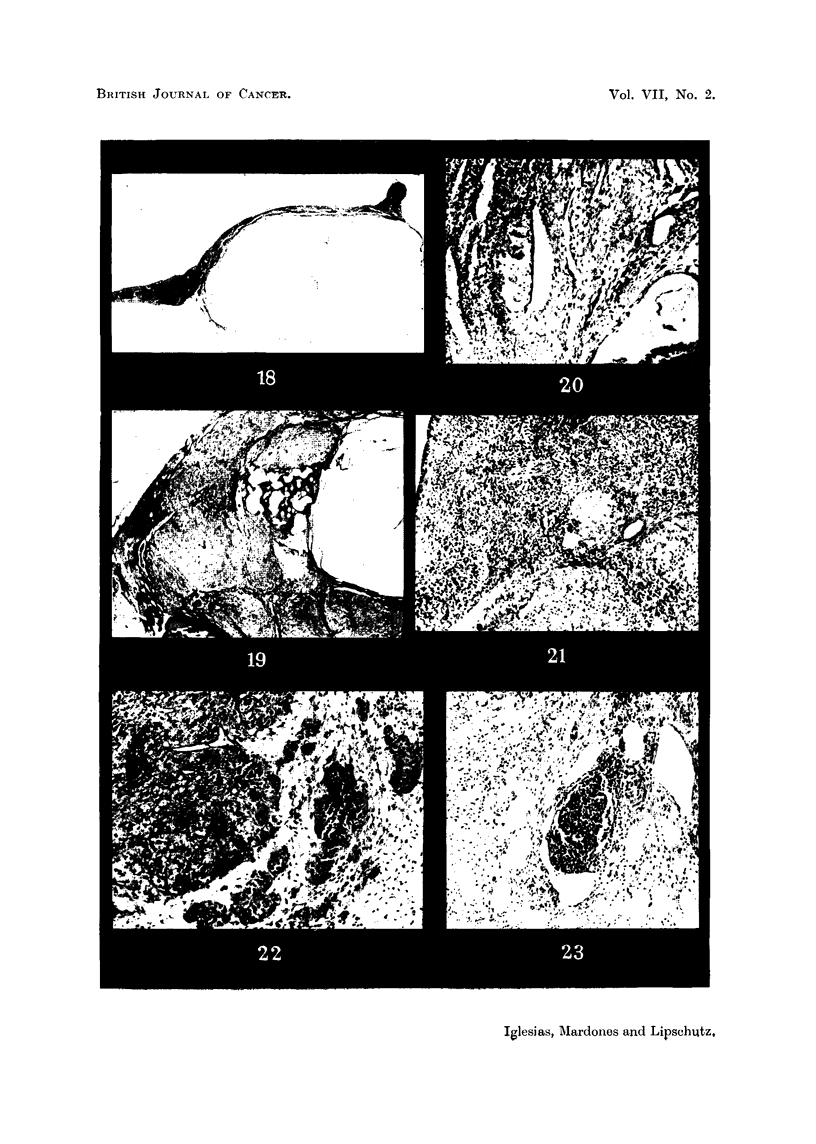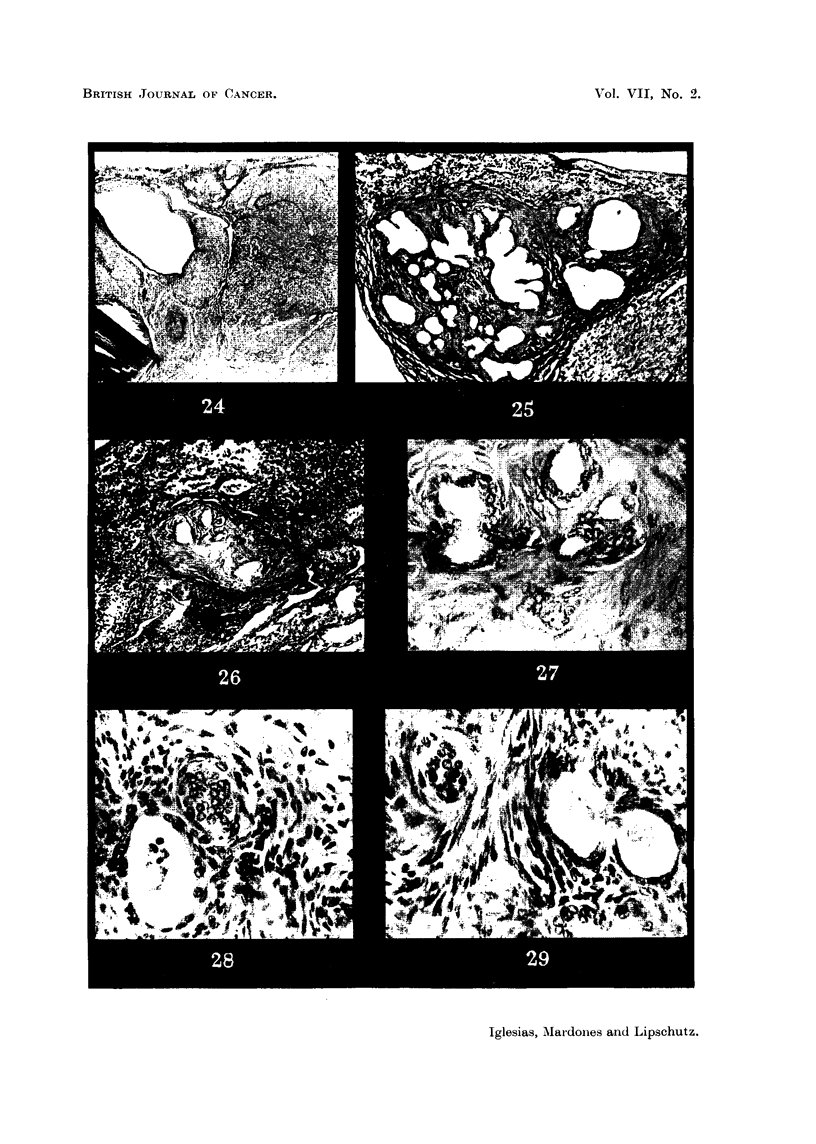# Evolution of Luteoma in Intrasplenic Ovarian Grafts in the Guinea-Pig

**DOI:** 10.1038/bjc.1953.19

**Published:** 1953-06

**Authors:** R. Iglesias, E. Mardones, A. Lipschutz

## Abstract

**Images:**


### EVOLUTION OF LUTEOMA IN INTRASPLENIC OVARIAN GRAFTS IN THE GUINEA-PIG.

R. IGLESIAS, E. MARDONES, AND A. LIPSCHUTZ.

*From Departmento de Medicina Experimental, Servicio Nacional de Salud, Avenida Irarrâzaval 849, Santiago de Chile.*

Received for publication February 2, 1953.

THE behaviour of the intrasplenic ovarian graft in the castrated guinea-pig has been studied in this Department since 1942. The graft shows two particularities : blood follicles which may appear as early as 3 weeks after transplantation and at 2 months are frequently cystically enlarged ; and small nodules and cords of luteal cells, i.e., of cells which resemble those of corpora lutea, scattered in the ovarian stroma. Later cn, at say 10 months, the graft consists, predominantly, of very large corpora lutea embedded in irregularly shaped masses of cells similar to those of corpora lutea (Ponce de León, 1944 ; Woywood, 1944 ; Lipschutz, 1946 ; Lipschutz, Ponce de León, Woywood and Gay, 1946 ; Iglesias, Lipschutz and Mardones, 1950). The condition has been termed by us as *luteomatous*. The luteal cells originate partly from small follicles whose theca—in some cases, but in a minor degree, possibly also the granulosa—undergoes precocious luteinization, and partly, according to our evidence, from cells of the stroma. Blood follicles and Graafian follicles were still present at 10 and even 20 months.

On the contrary, blood follicles fail to appear in the intrasplenic graft when the second ovary is left *in situ* (Gay, 1944) or grafted into the kidney (Ramírez, 1950). They appear only exceptionally when a minute ovarian fragment is left *in situ* (Gay, 1944 ; Niedmann, 1947 ; Bruzzone, Lipschutz and Niedmann, 1952), whereas they invariably appear when both ovaries are grafted into the spleen (Ramírez, 1950 ; Ramírez, Iglesias, Mardones and Lipschutz, 1952). There is thus full evidence that the whole gamut of atypical changes, including tumourigenesis, is due to the hypophysial gonadotrophic activity not duly controlled by ovarian hormones, the latter having been inactivated to a considerable degree in the liver.

The luteomatous or tumoural structure described in the guinea-pig is different from that in other laboratory animals. Granulosa-cell tumours and luteomata appear in the intrasplenic graft in castrated rats (Biskind and Biskind, 1944, 1949 ; van Lancker and Maisin, 1950 ; Lacour, Oberling and Guérin, 1951). In mice granulosa-cell tumours are more prevalent than luteoma (Li and Gardner, 1947*a*, 1947*b* ; Li, 1948 ; Furth and Sobel, 1947). Metastases may occur in mice and the tumour is transplantable (Li, 1948). But transplantation may also fail (Furth and Sobel, 1947). In our work with the guinea-pig metastases have never been observed. Granulosa-cell tumours largely luteinized have been produced also in the rabbit (Peckham, Greene and Jeffries, 1948 ; Peckham and Greene, 1952).

In the present paper results are recorded which were obtained in the guinea-pig in experiments in which the intrasplenic ovarian graft has been examined 20

months up to more than 3 years after transplantation. Results shall be discussed with reference to the following relevant point: is the luteoma of the guinea-pig liable to change, in the course of time, as to its biological behaviour, in such a way that there might be a chance for inducing transplantable and metastasizing ovarian tumours in experiments lasting as long as 3 years?

### RESULTS.

### A. *Incidence of luteoma.*

As already stated, at say 10 months almost the whole ovarian stroma may be replaced by luteinized cells. But more often luteal cords or nodules are less massive and the corpora lutea are embedded in the loose and often oedematous ovarian stroma. It is only in the first case that we speak of luteoma. The difference between what we call "luteoma" and "non-luteoma" is only one of degree.

TABLE I.—*Intrasplenic Ovarian Autografts in 21 Castrated Guinea-pigs, 20 to 36 months after transplantation.*

| Group. | Duration (days). | Total number of animals.* | Animals with haemorrhagic follicles. | Animals with luteoma. | Animals with luteomatous invasion. | Animals with large cysts predominating. | Animals with "Brenner." |
|---|---|---|---|---|---|---|---|
| I† | 608 to 612 | 5 | 3 | 4 | 1 | 1 | 2 |
| II | 802 to 915 | 7 | 2 | 4 | 3 | 0 | 0 |
| III | 997 to 1093 | 9** | 5 | 5 | 5 | 1 | 1 |

* Animals omitted in which there were adhesions between spleen and abdominal wall with suppression of haemorrhagic follicles and growth of uterus. But see also exceptions in next note.

** Including 3 animals with adhesions between spleen and abdominal wall. There was in 2 cases growth of nipples and uterine growth. *But haemorrhagic follicles were present in all the* 3 *cases.* In one of these animals there was a luteoma.

† In the animals of this group tiny pellets containing 40 per cent. of progesterone were implanted into the ovary to be grafted, according to the technique of Iglesias (Iglesias, Lipschutz and Rojas, 1950), to study the question whether progesterone will act locally as an antiluteinizer, i.e., when not allowed to reach the general circulation. As seen from the table, 4 out of 5 animals had luteoma, notwithstanding the progesterone pellet in the graft.

As seen from Table I, at 20 months the number of animals with luteoma as defined above was very considerable. Various new structural aspects were discovered in the present series of long duration: (1) The luteoma may become "exclusive," or almost "exclusive"; (*a*) luteinized tissue becomes so overwhelming that Graafian follicles are absent or found only when a systematic search in a complete series is made (Fig. 5); (*b*) the whole ovary may be occupied by luteal tissue without individual corpora lutea being recognizable. Three grafts of 20, 30 and 33 months were especially impressive as to this (Fig. 5 and 6; 14 and 15; compare with Fig. 1 and 2). These grafts without individual corpora lutea are, in the fresh section, of an intense yellow colour. (2) The size of the luteomatous ovaries, especially of those with an exclusive luteoma, may become very considerable (Fig. 5 and 13). The greatest weight of the spleen with the ovary within was 5·8 g.; since the weight of the spleen of 10 animals of 640 to 1040 g. body-weight was between 0·8 and 1·2 g., with an average of 0·9 g., the graft must have reached a weight of about 5 g. (Fig. 13 and 14). This is 50 to 100 times the weight of a normal ovary in the guinea-pig In other cases the great size of the graft was due to Wolffian cysts (Fig. 18). (3) Tubular structures of Wolffian origin may be found in immediate contact with luteal tissue (Fig. 19); sometimes one doubts whether these structures are Wolffian or follicular (Fig. 2 and 3).

B. *Invasive growth of luteoma.*

The graft is surrounded by a capsule of fibrous tissue of variable thickness. The germinal epithelium is absent, unless the ovary has been grafted together with the thin capsule by which the organ is normally covered; the fibrous capsule of the graft is then, so to say, projected outside the ovary itself.

In the present experiments of long duration luteal tissue located outside the thick fibrous capsule attracted attention. Finally, from 20 months onwards, all luteomata become invasive (Table I; Fig. 8 and 9). However, invasion may occur already at 10 months, but without reaching, seemingly, the same degree as in experiments of longer duration.

Immediate contact of luteinized cells with the spleen is established through rupture of the capsule (Fig. 10 and 11). The areas of luteal tissue in immediate contact with the spleen are sometimes so extensive (Fig. 8) that one cannot but admit that successive proliferation of extracapsular luteal tissue takes place.

The large luteoma pictured in Fig. 13 and 14 (915 days) is of especial interest here. Two distinct parts can be easily recognized: one consisting of more or less compact luteal tissue surrounded by a fibrous capsule, the other consisting of loose luteal tissue. It would be difficult to explain the special aspect offered by this graft. The two bodies of luteal tissue are separated from one another by thick fibrous tissue in which there is a cleft (Fig. 15) resembling that which is observed when the ovary is grafted together with its normal capsule; but the epithelium, or endothelium, covering this cleft at some places did not allow any definite statement as to its origin. However, there is the fact that the capsule may very exceptionally show a strange metaplastic transformation as in Fig. 20, a condition observed but once. Is it possible that the unique picture offered by Fig. 15—the graft divided into two halves—could be due to proliferation of capsular endothelium?

In several cases we found a zone of necrotic appearance between the fibrous capsule of the graft and the splenic tissue, possibly due to luteal tissue invading the spleen and undergoing necrosis after a period of more or less abundant proliferation.

Rupture of the capsule is possibly not the only route by which invasion is affected. In Fig. 21 a small nodule of luteal tissue is seen outside the capsule. The picture is suggestive of luteal cells having migrated from the graft without rupturing the capsule. However, sections were not serial, and the nodule might have been but a part of a larger area of luteal tissue as in Fig. 12 where rupture of the capsule had occurred.

A picture like Fig. 10 can easily be understood as a sequel of invasion of luteal cells into the splenic pulpa, which thus may become engorged by luteal tissue. In Fig. 23 what seems to be splenic tissue was found in the loose part of the ovarian stroma which corresponds to the hilus of the ovary.

We have not seen metastases in our work in the guinea-pig. But it seems that they are a rare occurrence also in other species; they have been reported in mice, but seemingly not in the rat.

As already stated at the beginning of this paper, the terms " luteal " and " luteoma " have been used because the cells of which the tissue in question is composed resemble those of corpora lutea. But Fig. 4, 9 and 10 show that there is a considerable variation of types of cells which can be found in these luteomata. It would have been possible to add many other striking graphic examples. The cytological problem deserves certainly greater attention than that given in our work ; however, our group is not competent in this special field.

C. *Wolffian elements.*

The growth of Wolffian elements is stimulated in the intrasplenic graft. There is formation of cysts in the ovarian hilus ; the epithelium of the tubules may become ciliated ; fibro-adenomatous nodules comparable to, though not identical with, certain types of the Brenner tumour in women may be found. Various examples are given in Fig. 24 to 29 (Lipschutz, 1950, p. 224 ; Iglesias, Mardones, Bruzzone and Lipschutz, 1953).

D. *Experiments with transplantation of luteoma.*

Transplantation experiments were performed with 11 grafts aged 802 to 1092 days. A piece of about 10 mm.$^3$ of 5 grafts was transplanted into the spleen, and of 6 grafts into the brain, of a total of 35 animals. The transplants were examined 61 to 116 days later.

Of an especial interest are the results with 4 grafts which were recognized as being luteoma belonging to the age Groups II and III (Table I), including an " exclusive " luteoma. In none of the 16 animals with these transplants (12 intrasplenic, 4 intracerebral) was luteomatous tissue found. There was only a scar with necrotic tissue, possibly also some lutein cells with pyknotic nuclei.

Results were negative also with the remaining transplants of grafts containing luteal cords, corpora lutea, or a fibro-adenomatous Wolffian nodule (" Brenner ").

It must be borne in mind that failure of transplants to take may be due to the fact that in the guinea-pig we are working so far with highly hybridized strains.

### DISCUSSION.

In none of the 21 experiments of Table I was a structure similar to a granulosa-cell tumour of whatever type to be found. One may venture the idea that absence of granulosa-cell tumours might be explained by intervening luteinization of proliferated granulosa cells. Indeed, when luteinization is totally inhibited by the administration of progesterone a considerable part of the ovary is occupied by " follicular clusters " ; but these clusters are very similar to small atretic follicles—they are composed of small cells originating seemingly from the theca (Iglesias, Lipschutz and Mardones, 1950). Thus the conclusion is reached that the luteoma of the intrasplenic ovarian graft in the castrated guinea-pig is not a luteinized granulosa-cell tumour but a tumour *sui generis*, invasive but probably not metastasizing, and originating through precocious luteinization preferably of theca cells and of cells of the stroma (Fig. 22), though luteinization of proliferated granulosa cells and incorporation of corpora lutea losing their individuality (Fig. 6 and 15) may possibly also contribute to the production of the tumour.

As to the term " luteoma " used throughout this paper we may refer to the best known summaries on ovarian tumours in women. In the human being there seems to be no such alternative as " granulosa-cell tumour " or " theca-cell tumour " versus " luteoma ". Ewing (1940, p. 658) admits that a " genuine " luteoma arising from the adult cells of corpora lutea may occur ; but he holds the opinion that marked luteinization may take place in granulosa-cell tumours, in such a way that " many, if not most, of the luteomas are really granulosa-cell tumours ". And likewise theca-cell tumours may, thanks to luteinization, resemble " true " luteoma. The last-mentioned opinion is held also by Geist (1942, p. 295) : " the lutein-cell tumour, if it may be classed as a separate unity, probably represents a luteinization of a granulosa- or theca-cell tumour ". According to Novak (1948, p. 458) " the common source of luteoma is through luteinization of granulosa-cell or theca-cell tumours ". Again, Willis (1948, p. 496) makes the statement that " luteal cell structure . . . is frequent in parts of granulosa-cell or theca-cell tumours, and is sometimes predominant, giving the tumour a bright yellow colour like a giant corpus luteum ". All authorities insist in the eventuality of luteinization of granulosa-cell tumours (Selye, 1946 ; Foot, 1948 ; Schiller, 1948 ; Moore, 1951).

From the point of view of comparative pathology one may formulate the differences between guinea-pig on one hand, mouse and rat on the other hand, in the following two statements : *first*, there is, in the guinea-pig, under the given abnormal hypophyseal conditions, possibly a more pronounced tendency to proliferation of theca cells than of granulosa cells ; and *secondly*, luteinization of theca cells and of cells of the stroma is, in the guinea-pig, more frequent and more prevalent than in the mouse, whereas the rat occupies an intermediate position between the other two species.

### SUMMARY.

1. Experiments with intrasplenic ovarian autografts in castrated female guinea-pigs lasting up to 3 years are reported, and the evolution of luteomata in these grafts is discussed.

2. The experimental luteoma of the guinea-pig is defined as a growth consisting of cords and nodules of luteinized cells of the stroma and of precociously luteinized cells of the theca of small follicles and in a minor degree possibly also of the granulosa, the masses of lutein cells occupying the greater part of the ovary.

3. Incidence of similar luteomata is, at 10 to 36 months after transplantation, considerable and reaches about 60 per cent.

4. In some cases follicles disappear and individual corpora lutea are lacking ; almost the whole ovary consists then of confluent cords and nodules of lutein cells (" exclusive " luteoma). Possibly individual corpora lutea, reaching an enormous size, lose their individuality and become incorporated into the luteoma.

5. Such an " exclusive " luteoma may reach a weight up to 50 to 100 times that of a normal ovary.

6. The longer the experiment lasts the greater is the chance that luteal tissue will be found outside the fibrous capsule which surrounds the graft, in immediate contact with splenic tissue.

7. The invasion of the spleen by luteal tissue takes place through rupture of the fibrous capsule, but possibly also without rupture.

8. There were no metastases.

9. The luteoma did not survive when transplanted into the spleen or the brain of other castrated guinea-pigs. This may be due to the high degree of hybridization in our guinea-pigs.

### REFERENCES.

BISKIND, G. R., AND BISKIND, M. S.—(1949) *Amer. J. clin. Path.*, **19**, 501.

BISKIND, M. S., AND BISKIND, G. R.—(1944) *Proc. Soc. exp. Biol., N.Y.*, **55**, 176.

BRUZZONE, S., LIPSCHUTZ, A., AND NIEDMANN, L.—(1952) *J. Endocrin.*, **8**, 187.

EWING, J.—(1940) 'Neoplastic Diseases.' 4th ed. Philadelphia (Saunders).

FOOT, N. C.—(1948) 'Identification of Tumours.' Philadelphia (Lippincott).

FURTH, J., AND SOBEL, H.—(1947) *J. nat. Cancer Inst.*, **8**, 7.

GAY, O.—(1944) Tesis, Universidad de Chile (Public Dep. Med. Exp. No. 30).

GEIST, S. H.—(1942) 'Ovarian Tumours.' New York (Hoeber).

IGLESIAS, R., LIPSCHUTZ, A., AND MARDONES, E.—(1950) *J. Endocrin.*, **6**, 363.

*Iidem* AND ROJAS, G.—(1950) *Endocrinology*, **46**, 414.

*Idem*, MARDONES, E., BRUZZONE, S., AND LIPSCHUTZ, A.—(1953) *Arch. Anat. micr. Morph. exp.* (In press).

LACOUR, F., OBERLING, CH., AND GUÉRIN, M.—(1951) *Bull. Ass. franç. Étude Cancer*, **38**, 128.

LANCKER, J. VAN, AND MAISIN, J.—(1950) *Ve Congrès int. Cancer*, p. 37.

LI, M. H.—(1948) *Amer. J. Obstet. Gynec.*, **55**, 316.

*Idem* AND GARDNER, W. U.—(1947*a*) *Science*, **105**, 13.—(1947*b*) *Cancer Res.*, **7**, 549.

LIPSCHUTZ, A.—(1946) *Nature*, **157**, 551.—(1950) 'Steroid Hormones and Tumours.' Baltimore (Williams & Wilkins).

*Idem*, PONCE DE LEÓN, H., WOYWOOD, E., AND GAY, O.—(1946) *Rev. canad. Biol.*, **5**, 181.

MOORE, R. A.—(1951) 'Textbook of Pathology.' 2nd ed. Philadelphia (Saunders).

NIEDMANN, L.—(1947) Tesis Universidad de Chile (Public. Dep. Med. Exp. No. 56).

NOVAK, E.—(1948) 'Textbook of Gynecology.' Baltimore (Williams & Wilkins).

PECKHAM, B. M., AND GREENE, R. R.—(1952) *Cancer Res.*, **12**, 654.

*Iidem* AND JEFFRIES, M. E.—(1948) *Science*, **107**, 319.

PONCE DE LEÓN, H.—(1944) Tesis, Universidad de Chile (Public. Dep. Med. Exp. No. 25).

RAMIREZ, H.—(1950) Tesis, Universidad de Chile (Public. Dep. Med. Exp. No. 77).

*Idem*, IGLESIAS, R., MARDONES, E., AND LIPSCHUTZ, A.—(1952) *Bol. Soc. Biol. (Santiago)*. (In press.)

SCHILLER, W.—(1948) *In* Anderson, W. A., Ee. 'Pathology.' St. Louis (Mosby), p. 1106.

SELYE, H.—(1946) 'Ovarian Tumours.' Montreal (Acta Inc.).

WILLIS, R. A.—(1948) 'Pathology of Tumours.' London (Butterworth).

WOYWOOD, E.—(1944) Tesis, Universidad de Chile (Public. Dep. Med. Exp. No. 26).

### EXPLANATION OF PLATES.

FIG. 1.—Luteoma in intrasplenic ovarian graft; 612 days (cxxxv.111). Individual corpora lutea and large irregularly shaped masses of luteinized cells. The cavity occupying almost the center belongs to a progesterone pellet (see † in Table I). The cavity beneath is Wolffian. The smaller cavity to the right is the cystic part of a system of tubular structures probably Wolffian. × 5.

FIG. 2.—Same animal as Fig. 1. Luteal bodies surrounding clefts. Irregularly shaped luteal masses to the right of the pellet cavity. × 15.

FIG. 3.—Same animal as Fig. 1 and 2. Luteal masses surrounding the clefts at the upper right of Fig. 2. Between the cleft and the luteal masses, hyalinized tissue. The epithelium is rather Wolffian but this is not certain. × 70.

Fig. 4.—Same animal as Fig. 1. Luteal cells of different types. Tubular structure at the upper left. × 300.

Fig. 5.—" Exclusive " luteoma in intrasplenic ovarian graft; 612 days (cxxxv.112). Individual corpora lutea are absent, the ovary consisting almost exclusively of a continuous mass of luteal cells. The whole graft was cut into serial sections and only one small Graafian follicle was found.

Fig. 6.—Same animal as Fig. 5. Compare Fig. 2. × 15.

Fig. 7.—Same animal as Fig. 5. Luteal cells of which the graft is composed. × 70.

Fig. 8.—Same animal as Fig. 5. Enormous mass of luteal cells in immediate contact with the spleen. × 70.

Fig. 9.—Same animal as Fig. 5. Cords and nodules of luteal cells in immediate contact with the spleen. × 100.

Fig. 10.—Same animal as Fig. 5. Luteal masses in immediate contact with spleen and engorging splenic tissue. × 70.

Fig. 11.—Same animal as Fig. 5. Rupture of the fibrous capsule surrounding the graft. Luteal masses taking direct contact with spleen. × 70.

Fig. 12.—This figure is the continuation of Fig. 11. Luteal cells between fibrous capsule and spleen. × 70.

Fig. 13.—Intrasplenic ovarian graft transformed into " exclusive " luteoma; 915 days (CLI.43). Spleen with graft. Natural size.

Fig. 14.—Same animal as Fig. 13. Section. Macrofoto. This luteoma differs from that of Fig. 5 in so far as the large luteal masses are separated from one another by thin fibrous trabecles. " Exclusive *lobulated* luteoma " would probably be the best term for this type of luteoma. Two different halves of the growth are easily distinguishable. The right one is seemingly compact, the left one is loose. × 3.

Fig. 15.—Same animal as Fig. 13. The large cleft between the two halves of the growth is an artefact due to fixation but part of it is of capsular origin. × 3.

Fig. 16.—Luteal tissue of the " loose " half of Fig. 15. × 300.

Fig. 17.—Luteal tissue of the " compact " half of Fig. 15. × 300.

Fig. 18.—Large Wolffian cysts in intrasplenic ovarian graft; 1100 days (cxxxvi.194). No other ovarian structures. This animal was omitted from Table I on account of adhesions. × 3·5.

Fig. 19.—Fibroadenomatous Wolffian nodule in intrasplenic ovarian graft in immediate contact with cords of luteal cells; 1092 days (cxxxvi.201). Individual corpora lutea; nodules of luteal cells. Not counted as luteoma. Large cyst, probably follicular. × 15.

Fig. 20—Intrasplenic ovarian graft; 305 days (cxxvi.153). Not mentioned in Table I. Haemorrhagic follicles, right bottom corner. Cleft due to ovarian capsule. Large cells in immediate contact with spleen. × 70.

Fig. 21.—Invasion of spleen by luteal masses of an intrasplenic ovarian graft with luteoma; 802 days (cxxvi.A). × 70.

Fig. 22.—Luteoma in intrasplenic ovarian graft; 612 days (cxxxv.114). Large masses of luteal cells and luteal cords in the loose oedematous ovarian stroma. × 70.

Fig. 23.—Same animal as Fig. 22. Nodule of small cells in the stroma, probably from the spleen. × 70.

Fig. 24.—Fibroadenomatous Wolffian nodule in intrasplenic ovarian graft with luteoma; 303 days (cxxvi.146). Not mentioned in Table I. The nodule is between the border of the ovary and the spleen; it is surrounded by luteal tissue. To the left a cyst of Wolffian origin. × 15.

Fig. 25.—Same animal as Fig. 24. The fibroadenomatous nodule at a higher magnification. The enlarged tubules are embedded in dense fibrous tissue. × 70.

Fig. 26.—Small fibroadenomatous Wolffian nodule in intrasplenic ovarian graft with luteoma; 305 days (cxxvi.158). Not mentioned in Table I. The nodule protrudes from the ovarian stroma into the spleen. Dense fibrous tissue prevails. × 70.

Fig. 27.—Same animal as Fig. 26. " Walthard " bodies (?) embedded in dense fibrous tissue probably undergoing hyalinization. × 300.

Fig. 28.—Same animal as Fig. 22. " Walthard " bodies (?) of a fibroadenomatous Wolffian nodule present in this animal. × 300.

Fig. 29.—Same as Fig. 28. × 300.

1

2

4

3

5

6

Iglesias, Mardones and Lipschutz.

Iglesias, Mardones and Lipschutz.

13

14

16

17

15

Iglesias, Mardones and Lipschutz.

Iglesias, Mardones and Lipschutz.